# Wet beriberi in intestinal failure due to change from intravenous to enteral supplementation – Time to focus on water-soluble vitamins

**DOI:** 10.1016/j.intf.2026.100372

**Published:** 2026-04-28

**Authors:** Benjamin Loel, Julia Fox, Katie Zajac, Looi Ee, Jonathan Hind

**Affiliations:** a Gastroenterology, Hepatology and Liver Transplant Service, Queensland Children's Hospital, Brisbane, Australia; b Dietetics Department, Queensland Children's Hospital, Brisbane, Australia

**Keywords:** Parenteral nutrition, Thiamine deficiency, Beriberi

## Abstract

**Background:**

Thiamine deficiency is a rare but life-threatening complication for which children on long term parenteral nutrition (PN) are at higher risk.

**Case report:**

We describe a 13-year-old male with intestinal failure secondary to Filamin A mutation who presented with one-week of oedema and orthopnoea. He was fully dependent on PN and his intravenous multivitamin supplementation was replaced with enteral vitamins due to a national shortage. On presentation he was tachycardic, tachypnoeic, hypotensive, and fluid overloaded. Investigations revealed hyponatraemia, hypoalbuminaemia, metabolic acidosis, and a significantly elevated lactate. Chest radiography showed bilateral pleural effusions. Intravenous thiamine and oral vitamin C supplementation led to rapid clinical improvement and testing confirmed low thiamine and vitamin C levels. His presentation was consistent with wet beriberi due to thiamine deficiency.

**Conclusion:**

This case highlights the risk of water-soluble vitamin deficiency in PN-dependent children during intravenous vitamin shortages and the need for biochemical monitoring.

## Introduction

Thiamine (vitamin B1) is a water-soluble vitamin essential for carbohydrate metabolism, myocardial energy production and neurological function. Deficiency results in impaired oxidative metabolism, lactic acidosis, peripheral vasodilatation and high-output cardiac failure, classically termed wet beriberi.

Children dependent on long-term parenteral nutrition (PN) are particularly vulnerable. Body thiamine stores are limited and since enteral intake and/or absorption is often inadequate, micronutrient provision relies entirely on PN composition. Disruptions to multivitamin injection (MVI) supply chains therefore pose a substantial risk. We present a case of life-threatening thiamine deficiency in a PN-dependent child following a transition from intravenous to enteral vitamin supplementation during a period of MVI shortage.

## Case report

A 13-year-old male presented with one week of cough, progressive breathlessness, lethargy, and generalised swelling. He had no fever, infective symptoms, or change in bowel or urinary habits.

His medical history was significant for intestinal failure due to gastrointestinal dysmotility associated with Filamin A mutation. His other relevant medical history included joint hypermobility, aortic root dilatation with aortic valve repair, scoliosis, previous basilic vein thrombus, and autism spectrum disorder. He had previously undergone Ladd’s procedure for intestinal malrotation.

He was dependent on home PN for 14 h daily via a tunnelled Broviac catheter and had negligible enteral intake because of marked oral aversion and dysmotility. He was however on oral medications including warfarin with acceptable anticoagulation. Five and a half months prior to presentation, his PN formulation was changed to a non-vitamin containing formulation in response to a national shortage of intravenous water-soluble MVI (Soluvit). His fat- and water-soluble vitamins were administered enterally in the form of VitABDECK multivitamin tablets 6 day/week, with comparable dosing of fat- and water-soluble vitamins when compared to his previous intravenous formulations (Table 1). His ability to absorb enteral vitamins was anticipated since his underlying disease was dysmotility and he was able to absorb oral warfarin and achieve stable anticoagulation.

**Table 1:** Comparison of vitamin doses between Intravenous and enteral supplementation.

**Vitamins**	**IV dose**	**Enteral dose**
Vitamin A (IU)	10960	15000
Vitamin D (mcg)	48	66
Vitamin E (IU)	33	900
Vitamin K (ug)	953	900
Thiamine (mg)	15	9
Riboflavin (mg)	23	10.2
Niacin (mg)	191	120
Pantothenic acid (mg)	79	72
Pyridoxine (mg)	23	11.4
Vitamin B12 (ug)	24	18
Vitamin C (mg)	538	600
Biotin (ug)	286	600
Folic acid (ug)	1906	1200

On examination, he was pale and oedematous with prominent periorbital swelling and peripheral oedema affecting all limbs. His weight was 47.7 kg, an increase of approximately 8 kg compared to his weight nine days earlier. He was tachycardic (130–132 beats/min), tachypnoeic (32–35 breaths/min) with cool cyanotic peripheries but normal oxygen saturations. Breath sounds were reduced at both lung bases, and a loud systolic murmur consistent with known aortic valve disease was present. His abdomen was distended but soft with no organomegaly and had generalised oedema which was non-pitting in the upper limbs and pitting in his lower limbs.

Initial investigations revealed marked hyponatraemia (124 mmol/L), hypoalbuminaemia (25 g/L), and compensated metabolic acidosis with an elevated lactate of 6.6 mmol/L. Renal and liver function were preserved. BNP was moderately elevated, while troponin and procalcitonin were normal (Table 2). Chest radiography demonstrated new bilateral pleural effusions, and abdominal ultrasound showed features of fluid overload, including a dilated inferior vena cava, without hepatic or portal pathology. Echocardiography confirmed stable valvular disease with preserved biventricular systolic function.

**Table 2:** Results of initial blood tests taken at time of presentation.

**Test**	**Result**	**Reference Range**
Haemoglobin	116 g/L	115–155 g/L
White cell count	17.2 × 10⁹/L	4.5–13.5 × 10⁹/L
Neutrophils	10.2 × 10⁹/L	1.5–8.0 × 10⁹/L
Platelets	116 × 10⁹/L	150–400 × 10⁹/L
INR	**4.1**	0.9–1.2 (therapeutic on warfarin 2–3)
**Test**	**Result**	**Reference Range**
Sodium	**124 mmol/L**	135–145 mmol/L
Potassium	4.3 mmol/L	3.5–5.0 mmol/L
Chloride	97 mmol/L	98–107 mmol/L
Bicarbonate	**16 mmol/L**	22–28 mmol/L
Urea	10.6 mmol/L	2.5–7.8 mmol/L
Creatinine	37 µmol/L	30–70 µmol/L
Serum osmolality	**270 mOsm/kg**	275–295 mOsm/kg
Albumin	**25 g/L**	35–50 g/L
Liver function tests	Normal	—
**Parameter**	**Result**	**Reference Range**
pH	7.35	7.32–7.43
pCO₂	31 mmHg	38–50 mmHg
Bicarbonate	**17 mmol/L**	22–28 mmol/L
Base excess	**−7.5 mmol/L**	−2 to + 2 mmol/L
Lactate	**6.6 mmol/L**	0.5–2.2 mmol/L
**Test**	**Result**	**Reference Range**
B-type natriuretic peptide (BNP)	**331 pg/mL**	< 100 pg/mL
Troponin	Normal	—
C-reactive protein (CRP)	**22 mg/L**	< 5 mg/L

He was initially treated with antibiotics, albumin and diuretics but rapidly deteriorated developing increased hypotension, oliguria, and worsening lactic acidosis. He was transferred to the paediatric intensive care unit for ionotropic support, diuresis and further evaluation. No infectious, renal, hepatic, or primary cardiac cause was identified to explain the severity of his presentation.

**Fig. 1:**
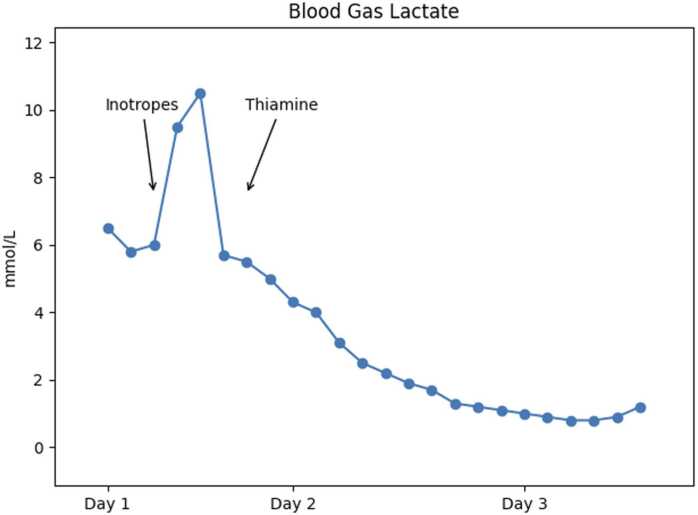
Serum lactate (mmol/L) during admission in relation to timing of intervention with ionotropes and intravenous thiamine (see arrows).

**Fig. 2:**
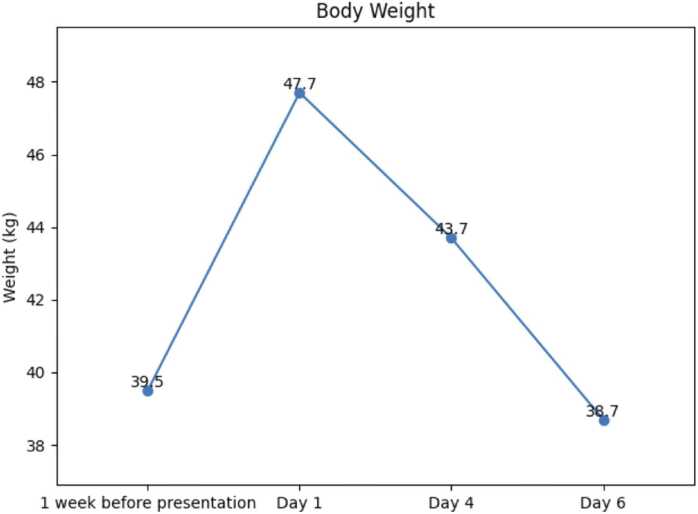
Graph depicting the patient’s body weight beginning 1 week prior to presentation (21/11/24) with subsequent weight gain at presentation which improved throughout the admission with diuresis.

Given his complete dependence on PN and recent formulation change, a nutritional deficiency was suspected. He was empirically treated with intravenous thiamine and oral vitamin C with rapid clinical improvement such that he was able to wean off ionotropic support overnight and tolerated a frusemide infusion for diuresis. He was treated with intravenous thiamine 100 mg twice daily for three days before reducing to once daily and oral vitamin C 1000 mg daily. Lactate levels normalised within twenty-four hours, and his weight normalised to his premorbid measurement by day three of admission (Fig. 1, Fig. 2). He was subsequently confirmed to have a critically low thiamine diphosphate level and a low vitamin C concentration (Table 3). His potassium, magnesium, trace elements including copper and selenium, and fat-soluble vitamin levels were normal, and stool alpha-1 antitrypsin excluded protein-losing enteropathy.

**Table 3:** Results of nutritional blood testing.

**Test**	**Result**	**Reference Range**
Thiamine diphosphate	**0.25 mmol/g Hb**	0.7–1.8 mmol/g Hb
Thiamine diphosphate (post-treatment)	1.9 mmol/g Hb	0.7–1.8 mmol/g Hb
Vitamin C	**5 µmol/L**	20–120 µmol/L
Fat-soluble vitamins (A, D, E, K)	Normal	—
Trace elements	Normal	—

Diuretics were discontinued by day six and his repeat thiamine levels returned to normal. He was discharged home clinically well eight days after initial presentation on 100 mg thiamine daily and 500 mg vitamin C twice daily, with a re-formulated PN regimen which included vitamins. Ongoing micronutrient surveillance was planned, and subsequent blood tests confirmed that he maintained normal levels at follow up.

## Discussion

Thiamine is an essential cofactor in oxidative carbohydrate metabolism. Deficiency disrupts the activity of pyruvate dehydrogenase and related mitochondrial enzymes, diverting pyruvate toward lactate production and resulting in type B lactic acidosis. The downstream consequences include peripheral vasodilatation, high-output cardiac failure (wet beriberi), and Wernicke-like encephalopathy, particularly affecting organs with high metabolic demand such as the heart and brain [1].

Children dependent on long-term PN are uniquely vulnerable to thiamine deficiency because enteral intake is often negligible, body stores are limited, and metabolic requirements are high. In this setting, micronutrient sufficiency depends entirely on the adequacy and route of vitamin provision within the PN formulation [2], [3]. When appropriately dosed paediatric MVI preparations are included daily, thiamine levels are generally maintained within the reference range, thus clinical deficiency is uncommon in both premature infants and older children [2], [4], [5], [6].

In contrast, omission, under-dosing, or rationing of MVIs has repeatedly been associated with severe thiamine deficiency in PN-dependent children. Numerous case reports describe preterm and term infants receiving unsupplemented or inadequately supplemented PN who developed profound lactic acidosis and metabolic collapse within weeks. In these cases, biochemical abnormalities and clinical instability resolved rapidly following intravenous thiamine administration [7], [8], [9], [10], [11], [12]. Similar presentations have been reported in older children receiving PN for malignancy, liver transplantation, or prolonged gut rest, again with dramatic responses to thiamine replacement [13], [14], [15].

National and regional shortages of MVIs have emerged as a key systemic contributor to these events. During a shortage in the United States in 1997, cases of lactic acidosis attributable to thiamine-deficient PN were reported, alongside instances of Wernicke encephalopathy and beriberi in PN-dependent patients [16], [17]. Subsequent reviews have linked PN additive shortages and rationing strategies in neonatal intensive care units to adverse outcomes, including metabolic acidosis and neurological injury [3]. More recent shortages have produced clusters of combined B-vitamin deficiencies (including B1, B2, B6, and biotin) in PN-dependent neonates [8], [18], [19], [20]. Another recent single centre study of home PN populations have also raised concern that oral multivitamin preparations may not reliably protect against thiamine deficiency during periods of MVI unavailability [21].

Thiamine deficiency has also been described following PN weaning in children with intestinal failure, where reliance on enteral vitamin supplementation proved insufficient and acute neurological presentations ensued [22].

The fat-soluble vitamin levels remained normal in our patient during the period of enteral replacement. This likely reflects the difference in physiologic storage and half-life of these vitamins when compared to water-soluble vitamins.

Taken together, these cases highlight an important and recurring pattern. PN-dependent children, particularly those with intestinal failure or complex gastrointestinal disease, remain at risk of thiamine deficiency not only when intravenous vitamins are omitted, but also during transitions from intravenous to enteral supplementation. Factors such as malabsorption, high gastrointestinal output, altered anatomy, and poor adherence all limit the reliability of enteral vitamin delivery in this population.

The existing literature supports three key themes relevant to the present case. First, life-threatening thiamine deficiency in PN-dependent children occurs almost exclusively when MVI is omitted, rationed, or ineffectively substituted, with wet beriberi and lactic acidosis representing characteristic and rapidly reversible phenotypes [7], [8], [9], [10], [11], [12]. Second, international and national shortages of intravenous MVI are well-described precipitants of such events and demand institutional strategies to ensure minimum supply and appropriate contingencies during periods of low stock [16], [17], [18], [20]. Finally, transitions from intravenous to enteral vitamin replacement in children with intestinal failure or long-term PN dependence must be undertaken cautiously. Enteral supplementation cannot be assumed to provide adequate thiamine status, and proactive monitoring of water-soluble vitamin levels combined with a low threshold for empiric parenteral supplementation is warranted in this high-risk group [21], [22].

In conclusion, thiamine deficiency is a rare but preventable cause of life-threatening lactic acidosis, cardiac failure, and neurological injury in PN-dependent children. In the era of standardised PN solutions, shortages may be inevitable. This case serves as an important reminder of the often-overlooked perils of water-soluble vitamin deficiencies.

## CRediT authorship contribution statement

**Jonathan Hind:** Writing – review & editing, Writing – original draft, Conceptualization. **Looi Ee:** Writing – review & editing, Writing – original draft, Conceptualization. **Katie Zajac:** Writing – review & editing, Writing – original draft. **Julia Fox:** Writing – review & editing, Writing – original draft, Conceptualization. **Benjamin Loel:** Writing – review & editing, Writing – original draft, Conceptualization.

## Declaration of generative AI and AI-assisted technologies in the manuscript preparation process

During the preparation of this work the authors used *Undermind AI, Inc.* in order to assist with a literature review and make final edits. After using this tool, the authors reviewed and edited the content as needed and take full responsibility for the content of the published article.

## Patient's/ Guardian's consent

The patient and patients parent provided consent to include the clinical information provided for publication.

## Patient's/ Guardian's consent

Obtained

## Ethical clearance

Not required

## Funding

This research did not receive any specific grant from funding agencies in the public, commercial or not-for-profit sectors.

## Declaration of Competing Interest

The authors declare that they have no known competing financial interests or personal relationships that could have appeared to influence the work reported in this paper.
